# Impact of metformin use on survival outcomes in non-small cell lung cancer treated with platinum

**DOI:** 10.1097/MD.0000000000013652

**Published:** 2018-12-21

**Authors:** Xin Wen-Xiu, Zheng Xiao-Wei, Ding Hai-Ying, Tong Ying-Hui, Kong Si-Si, Zhou Xiao-Fang, Ping Huang

**Affiliations:** a Laboratory of Clinical Pharmacy; b Key Laboratory of Head and Neck Translational Research of Zhejiang Province Zhejiang Cancer Hospital, Hangzhou, P.R. China.

**Keywords:** metformin, NSCLC, platinum, survival, T2DM

## Abstract

Preclinical evidence suggests that metformin, a widely used antidiabetic drug, may have a sensitizing effect on platinum. The purpose of this study was to evaluate the survival outcomes for non-small cell lung cancer (NSCLC) patients with type 2 diabetes mellitus (T2DM) using metformin during platinum-based chemotherapy.

The clinicopathological parameters and survival data of 75 NSCLC patients with T2DM from January 2008 to December 2011 were collected and analyzed retrospectively. Patients were divided into 2 groups: metformin exposure group (n = 27) and non-metformin group (patients using other hypoglycemic agents or no drug for controlling n = 48). Univariate and multivariate analyses were performed to assess the association of metformin usage with overall survival (OS).

Mean follow-up time was 58.7 months. The mean survival time was 36.74 months in the metformin group and 40.21 months in the non-metformin group. There was no significant difference in survival time between the 2 groups (*P* = .661). After adjusting gender, age, smoking status, tumor stage, tumor histology, and differentiation, multivariate analysis showed that metformin was not associated with the OS in NSCLC patients treated with concurrent platinum-based chemotherapy (hazard ratio: 1.071, 95% confidence interval: 0.577–1.986, *P* = .828).

Our results indicated that metformin exposure had no significant effect on OS in NSCLC patients treated with platinum-based chemotherapy. Further studies are warranted to evaluate whether metformin could affect the survival of NSCLC patients treated with platinum-based chemotherapy.

## 1 Introduction

Even though we have made great breakthrough in the diagnosis and therapy of lung cancer in the latest decades, it still leads to high cancer-related deaths with only 5-year survival rate of 10% to 15%.^[1,2]^ Non-small cell lung cancer (NSCLC) takes about 80% to 85% share hold in lung cancer patients and unfortunately, 65% patients with NSCLC are diagnosed with late-stage disease when curative therapy is no longer available. Thereby, chemoradiotherapy is still the primary method for lung cancer therapy.^[3,4]^ Platinum plays an anti-tumor role mainly by directly acting on DNA of tumor cells and forming additives, inhibiting DNA's unwinding and replication.^[5]^ The combined chemotherapy regimen based on platinum is still one of the main treatments for NSCLC.^[6]^ However, the benefit of chemotherapy was generally modest, and sometimes tumor cells develop resistance to platinum after a period of use. Therefore, it is urgent to explore a new strategy to improve the efficiency of platinum drugs.

The overall prevalence of diabetes was about 11.6% in the Chinese adult population,^[7]^ of which type 2 diabetes mellitus (T2DM) accounts for more than 90%. Fifteen percent to 20% cancer patients suffer from T2DM and the proportion is increasing.^[8]^ Metformin is the most widely prescribed oral hypoglycemic drug worldwide for the treatment of type 2 diabetes. Metformin can reduce plasma glucose levels by enhancing insulin sensitivity, benefiting patients with various insulin-resistant states, including impaired glucose tolerance, polycystic ovary syndrome, obesity, and metabolic syndrome.^[9,10]^ A large number of preclinical, epidemiological, and clinical studies have found that metformin has an antitumor effect and may affect the prognosis of patients with lung cancer and diabetes.^[11]^ Tan et al^[12]^ found that the patients with diabetes treated with metformin had a longer progression-free survival (PFS) and overall survival (OS), and the difference was statistically significant. Medairos et al^[13]^ found that metformin may be associated with improved PFS in patients with early-stage NSCLC, but has no effect on OS. Researchers also found that metformin can enhance the sensitivity of platinum drugs and reverse resistance.^[14,15]^ However, the effect of metformin on NSCLC patients undergoing platinum-based chemotherapy is unclear.

This article focuses on the association between metformin and clinical outcomes of NSCLC patients with T2DM treated with concurrent platinum-based chemotherapy.

## 2 Materials and methods

### 2.1 Study population

The research project was reviewed and approved by the Ethics Committee of Zhejiang Cancer Hospital. We confirmed that all methods were performed on the basis of the relevant guidelines and regulations. Information of the patients diagnosed with NSCLC was searched in internal medical information system from January 2008 to December 2011 in this study. Inclusion criteria were as follows:

(1) pathological or cytological diagnosis of NSCLC;
(2) T2DM;
(3) treated definitively with platinum-based chemotherapy;
(4) complete clinical data and follow-up data.

Exclusion criteria as follows:

(1) type 1 diabetes mellitus;
(2) patients also had other cancers besides NSCLC. Control subjects were matched to the corresponding cases based on age (within 5-year intervals).

Finally, a total of 75 NSCLC patients with T2DM and concurrent platinum-based chemotherapy were included. 27 patients took metformin for long-term control of diabetes (metformin group), and the others used different hypoglycemic agents or no drug for controlling (non-metformin group).

### 2.2 Data collection

The medical records of the patients were directly reviewed to reduce bias and collect clinicopathological parameters such as gender, age, smoking status, alcohol consumption, tumor-lymph node-metastasis (TNM) classification, tumor pathology, differentiation, OS, the regimens, and cycles of chemotherapy. All NSCLC patients’ TNM classification using for disease staging was determined on the basis of thoracic and abdominal CT scans, bronchoscopy, MRI imaging of the central nervous system (CNS), whole-body bone scan and/or 18F-labeled fluorodeoxyglucose positron emission tomography (PET) scan. The follow-up duration was from enrolment to the end of December 2016. All patients were followed at an interval of approximately 2 to 3 months. Total follow-up information was collected from clinic visit or from electronic communication methods such as text messaging, telephone and E-mail. The main follow-up information was the survival status of patients, treatment of cancer and control of diabetes. The OS was defined as time from the date of diagnosis to the date of death or last visit.

### 2.3 Statistical analyses

SPSS version 23.0 (SPSS, Chicago, IL) was used for the statistical analysis. Categorical variables were analyzed by a Chi-squared test. The Kaplan–Meier method was performed to delineate the actuarial survival curves for OS. Log-rank tests were used to compare outcomes. Cox regression was performed to compare survival of patients on metformin versus other medications adjusting for gender, age, smoking status, alcohol consumption, tumor stage, tumor histopathology, and differentiation. Furthermore, the univariate and multivariate Cox regression analysis was conducted to identify whether there was a difference in the OS of each group. Hazard ratios (HR) and 95% confidence interval (CI) were generated. Statistical significance was set at an alpha value of *P* <.05.

## 3 Results

### 3.1 Patient characteristics

In this study, a total of 75 NSCLC patients who have been treated with platinum-based chemotherapy in our hospital from January 2008 to December 2011 were included. Among the 75 patients, 27 patients were included in the metformin group, and the others were included in the non-metformin group. Follow-up period ended in December 2016 and no one was lost. Median follow-up time was 58.7 months. Clinical records of the 2 groups were collected, and the clinicopathological features were compared by the Chi-square test. There was no significant difference (*P* >.05) between the 2 groups in gender (male 6/27 vs 12/48), age stratification (≥60 years old 11/27 vs 21/48), alcohol consumption before diagnosis (with drinking history 12/27 vs 20/48), tumor stage (III–IV period 20/27 vs 27/48) and differentiation (poorly differentiated 15/27 vs 27/48). In addition, the non-metformin group had significantly more smoking patients than metformin group (*P* = .016), and the proportion of patients with squamous cell carcinoma in non-metformin group was higher (*P* = .024).

### 3.2 Metformin use and survival prognosis in NSCLC patients

**Figure 1:**
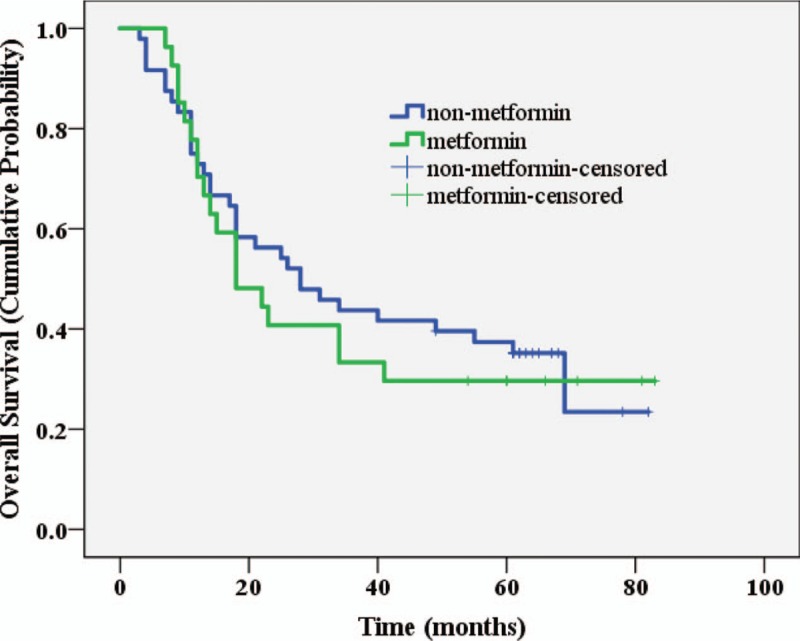
K–M plot of overall survival for the metformin users versus non-metformin users. K–M = Kaplan–Meier.

Twenty-five patients were still alive at the last follow-up on December 31, 2016, a total of 51 patients died, of which 32 in non-metformin group and 19 in metformin group. Detailed descriptive data for studies are presented in Table 1. Log-rank test was employed to analyze the differences in OS between the metformin group and the non-metformin group. Metformin group had a mean OS of 36.74 months with the 95% CI: 25.03–48.45 months and median survival time of 18 months with the 95% CI: 9.10–26.91 months. Non-metformin patients had mean OS of 40.21 months with the 95% CI: 31.34–49.08 months and median survival time of 28 months with the 95% CI: 13.30–42.70 months. The difference in survival was not statistically significant (*P* = 0.661) between the 2 groups. The survival times of the 2 groups are shown in Table 2, and the Kaplan Meier survival curve is shown in Figure 1.

**Table 1:**
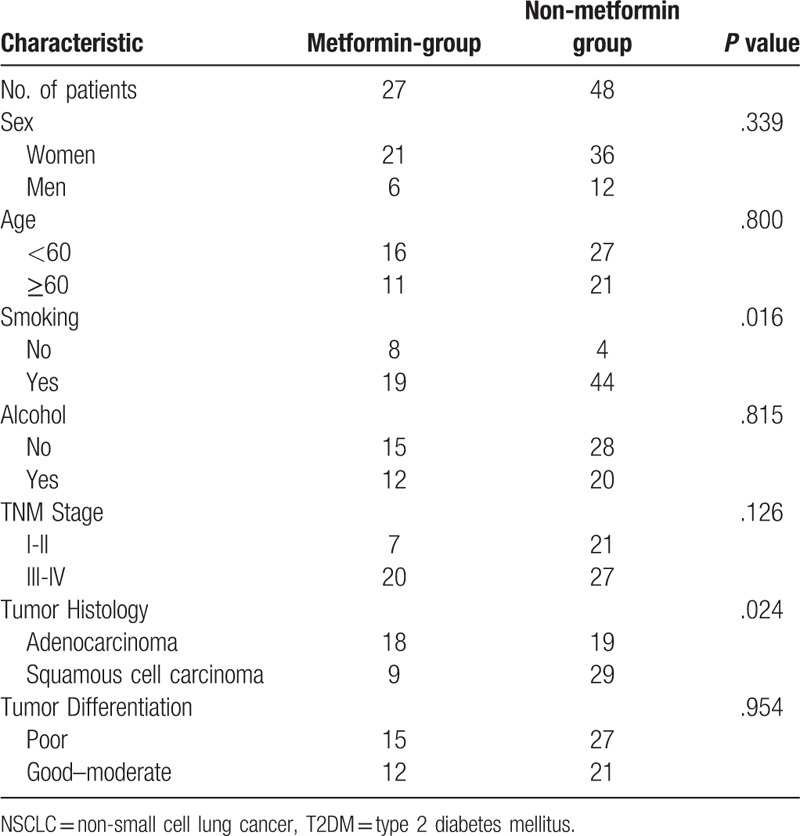
Clinicopathological characteristics of NSCLC patients with T2DM treated with platinum-based chemotherapy.

**Table 2:**
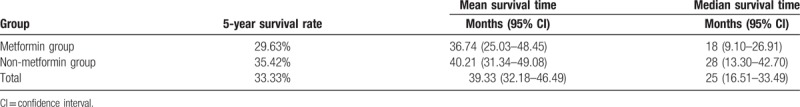
Comparison of survival time between metformin- and non-metformin-users.

Univariate and multivariate analysis found that the general poor OS of NSCLC patients with T2DM was mainly due to high TNM stage (*P* <.0001) as shown in Table 3. Considering that the factors such as gender, age, smoking status, alcohol consumption, tumor stage, histopathology, and differentiation may affect the patient's survival time, multivariate Cox regression analysis is performed. Results showed that after multiple factors correcting, metformin has no significant effect to the OS of NSCLC patients undergoing platinum-based chemotherapy (Hazard ratio: 1.071, 95% CI: 0.577 to 1.986, *P* = .828) (Table 3).

**Table 3:**
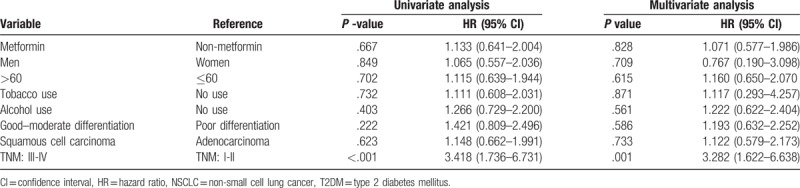
Hazard ratios for survival in NSCLC patients with T2DM treated with and without metformin.

## 4 Discussion

This study aimed at exploring the effect of metformin on the OS of NSCLC patients with T2DM during platinum-based chemotherapy. Both univariate and multifactor Cox regression showed that metformin exposure had no significant effect on the total survival of NSCLC patients with T2DM treated with platinum-based chemotherapy. The average and median survival time was 36.74 (25.03–48.45) months and 18 (9.10–26.91) months respectively in the metformin group. The average and median survival time was 40.21 (31.34–49.08) months and 28 (13.30–42.70) months respectively in the non-metformin group. There is no statistical difference between groups.

Diabetes and lung cancer both are diseases that have a high incidence worldwide. The frequency of NSCLC patients concurrent with diabetes is high. Epidemiological studies have shown that metformin can reduce the risk of cancer development and improve the prognosis of certain cancers, such as colorectal cancer,^[16]^ endometrial cancer,^[17]^ pancreatic cancer,^[18]^ and kidney cancer^[19]^ et al Observational studies have attempted to understand the effect of metformin on the prognosis of lung cancer. Some studies have suggested that the use of metformin is associated with an increased risk of death of lung cancer.^[20]^ However, other studies have shown that metformin benefits the survival of lung cancer patients^[21–25]^ or have no effect^[26,27]^. Due to the inconsistent results of epidemiological studies, several meta-analyses were carried out and the statistical results showed that the use of metformin could improve the survival of patients with lung cancer.^[28–31]^ In addition, various preclinical studies have found that metformin enhances the anti-tumor effects of platinum and increases their sensitivity by regulating multiple signaling pathways including AMPK-mTOR, ERK1/2, NF-κB.^[32–34]^ Thus, we hypothesized that metformin might benefit lung cancer patients who received platinum-based chemotherapy.

In retrospective epidemiological studies, potential for identifying associations related to sources of biases can never be overlooked. In order to reduce errors and biases, attempts were made by directly reviewing each study subjects’ medical records, by matching controls based on known risk factors, and by using multivariate analysis. An effect-cause error was avoided by insuring metformin use concurrent with platinum-based chemotherapy. However, this study shows that metformin has no significant effect on the OS of NSCLC patients treated with platinum-based chemotherapy. The inconsistent between the hypothesis and this study may lie in the following reasons: First, this study is single-center retrospective study with small sample size and lack of related clinical data, such as the KPS score, severity of diabetes, chemotherapy regimen, and radiotherapy. Therefore, it may have some bias in the results; In addition, both metformin and cisplatin need the same transporter organic cation transporter-2 (OCT2) to enter into the cell and play the role. Therefore, different from the isolated cell experiment, metformin, and cisplatin will compete for the same transporter in vivo, and then affect the anti-tumor efficacy.

In conclusion, our results indicated that the use of the biguanide metformin was not associated with a survival benefit in NSCLC patients treated with platinum-based chemotherapy. The full potential effect of metformin use on survival outcomes in NSCLC patients treated with platinum-based chemotherapy should be further rigorously accessed through randomized trials in the future.

## Author contributions

**Conceptualization:** Zheng Xiao-Wei.

**Data curation:** Zheng Xiao-Wei.

**Formal analysis:** Xin Wen-Xiu, Ding Hai-Ying.

**Investigation:** Tong Ying-Hui.

**Methodology:** Xin Wen-Xiu, Tong Ying-Hui.

**Software:** Xin Wen-Xiu.

**Supervision:** Ping Huang.

**Validation:** Kong Si-Si.

**Writing – original draft:** Xin Wen-Xiu.

**Writing – review & editing:** Zhou Xiao-Fang, Ping Huang.
